# Tackling Mantle Cell Lymphoma in Europe

**DOI:** 10.3390/healthcare10091682

**Published:** 2022-09-03

**Authors:** Denis Horgan, Jan Walewski, Igor Aurer, Carlo Visco, Eva Giné, Bogdan Fetica, Mats Jerkeman, Marta Kozaric, Maria Gomes da Silva, Martin Dreyling

**Affiliations:** 1 European Alliance for Personalised Medicine, 1040 Brussels, Belgium; 2 The Maria Sklodowska-Curie National Research Institute of Oncology, 00-001 Warszawa, Poland; 3 Division of Hematology, Department of Internal Medicine, University Hospital Center Zagreb, 10000 Zagreb, Croatia; 4 Department of Medicine, Section of Hematology, University of Verona, 37129 Verona, Italy; 5 Instituto Clínic de Enfermedades Hematológicas y Oncológicas, Hospital Clínic de Barcelona, 08036 Barcelona, Spain; 6 Department of Pathology, Institute of Oncology “Prof. Dr. Ion Chiricuta” Cluj-Napoca, 400015 Cluj-Napoca, Romania; 7 Department of Oncology, Institute of Clinical Sciences, Lund University and Skane, University Hospital, BMC F12, 221 84 Lund, Sweden; 8 Haematology Unit, Instituto Portugues de Oncologia de Lisboa Francisco Gentil, 1099-023 Lisbon, Portugal; 9 Medical Clinic III, Groβhadern Clinic, Ludwig-Maximilians-Universität, 81377 Munich, Germany

**Keywords:** mantle cell lymphoma, MCL, personalized medicine, healthcare, treatment, policy framework, care, infrastructure, barriers, EU level

## Abstract

An expert panel convened by the European Alliance for Personalized Medicine (EAPM) reflected on achievements and outstanding challenges in Europe in mantle cell lymphoma (MCL). Through the prism of member state experience, the panel noted advances in outcomes over the last decade, but highlighted issues constituting barriers to better care. The list notably included availability of newer treatments, infrastructure and funding for related testing, and shortages of relevant skills and of research support. The prospect of improvements was held to reside in closer coordination and cooperation within and between individual countries, and in changes in policy and scale of investment at both national and EU levels.

## 1. Introduction

Mantle cell lymphoma (MCL) is a hematological malignant disease composed of 2.5–6% non-Hodgkin’s lymphomas. The treatment has evolved over time, and different treatment options exist for patients with aggressive variants depending on age, performance status, and possibility of bone marrow transplant [1,2,3]. There are over 70 subtypes of lymphomas, with different diagnostic evaluation, different treatment protocols, and different outcomes. Lymphoma subtypes are often categorized into three major groups: chronic lymphocytic leukemia (CLL), Hodgkin lymphoma (HL), and non-Hodgkin lymphoma (NHL). Out of NHL, MCL is a very rare cancer of the B lymphocytes (B cells) [4,5]. This subtype of lymphoma is usually fast-growing, but it can also behave more indolently (slow-growing) in some patients [6]. Numerous advances in the understanding of the biology of MCL have been achieved over the years and consequently, there has been a rise in treatments and clinical trials with targeted therapies [7].

What can make tackling MCL more effective in Europe—and what makes it harder? Those are the questions addressed by an expert panel assembled by European Alliance for Personalized Medicine (EAPM) in early 2022, in a bid to improve the situation for patients at member state and EU level. Possibilities of successful treatment are improving, but there are still high unmet needs for patients including limited treatment options, wide variations in treatment regimens, availability, and continuing uncertainty over their use [8,9]. While research continues to illuminate the options, it can be impeded by lack of funding, as well as fragmentation of effort and deficiencies in the necessary infrastructure.

The discussions revealed that the challenges—including late or incorrect diagnosis, lack of access to appropriate therapies and expertise, lack of commercial feasibility in developing new therapies, difficulties in conducting well-powered clinical studies, and the paucity of tissue banks—could be meaningfully assessed by breaking them down into supply-side and demand-side issues. The demand side was seen as primarily influenced by governance, clinical standardization, awareness, and education, while the actual supply is influenced predominantly by equitable reimbursement, infrastructure for conducting and validating tests, and testing access driven by evidence generation. The principal conclusions to emerge included the desirability of linking national efforts by those engaged in care and promoting a European approach to reinforce improvements. Some of the gaps identified might, it was argued, be filled by assistance from the EU level, where current major policy initiatives offer direct or indirect opportunities. At the heart of the discussions was the need to find ways of persuading policy makers to support improved diagnosis and treatment of MCL patients. The picture drawn by the expert panel is of opportunities currently being missed, and potential unfulfilled. However, at the same time, there were ample suggestions of pathways towards seizing the opportunities and realizing the potential.

## 2. Situation Report

The current panorama of MCL care and research in Europe is exemplified by the reports and comments of experts at an EAPM panel in April 2022.

The picture of gaps in both demand and supply is unsatisfactory for patients, for clinicians, and for the future of effective therapy. Despite progress in treatment options, particularly in the last decade, MCL remains a disease with a high number of relapses and a poor long-term survival for many patients [9,10,11,12]. Though rare, it is an aggressive disease [13]. Although MCL generally responds to initial treatment, the disease inevitably relapses, and median overall survival following initial induction therapy is 3–5 years and exhibits varied clinical behavior and prognoses, reflecting the biologic heterogeneity of the disease [14,15,16,17]. Many recommendations regarding use of chemoimmunotherapy to treat relapsed/refractory MCL are based on limited studies and few randomized comparative trials [18]. Elucidation of the molecular pathophysiology of MCL has resulted in new and specifically targeted agents as alternatives to combination chemoimmunotherapy at diagnosis [19]. There are now possibilities to stratify patients as low, intermediate, or high risk [20]. However, although multiple treatment options are available as novel monotherapies for relapsed/refractory MCL, no clear standard of care is recognized in EU or US treatment guidelines [3,14]. Differences persist in the guidelines for induction therapy issued in the US and in Europe, and in recommended treatment regimens. Molecular-based agents together with chemoimmunotherapy may be appropriate for some patients but potential toxicity needs to be taken into consideration. Treatments differ with regards to efficacy, adverse event profiles and mode of administration, and the choice of treatment is also dependent on factors such as patient fitness and wishes [5,10,21]. The Mantle Cell Lymphoma International Prognostic Index (MIPI), the first prognostic index for MCL patients, is based on independent prognostic factors including age, Eastern Cooperative Oncology Group (ECOG) performance status, lactate dehydrogenase (LDH) level, and leucocyte count. On the other hand, Ki-67 staining by immunohistochemistry, which identifies proliferating cells, has showed strong prognostic relevance in MCL both alone and when combined with the MIPI [22]. A study conducted by the European MCL Network confirmed that the NanoString platform-based MCL35 assay is a reliable prognostic biomarker in MCL patients which identifies subgroups with different outcomes via a proliferation signature-based score [23]. Specialists have an important role, as in all rare cancers, to identify patients early and assess the most appropriate treatment based on clinical characteristics and personal choice of each patient [7,24,25].

Globally, incidence of NHL has increased while mortality and its annual percentage change have remained relatively stable [26]. According to the Munich Cancer Registry in Germany, for the period 2007–2020, average mortality of patients with MCL was 0.7/100,000 WS, N = 249 for males while for females it was 0.2/100,000 WS N = 111 [27]. In France, it was shown that when comparing patients with MCL included in clinical trials or registered in cancer registries, patients from clinical trials were younger and had a less advanced stage. An excessive mortality was seen mainly in elderly MCL patients from registries, confirming patient selection bias in clinical trials [28]. A Spanish study which involved 177 lymphoma patients with COVID-19 infection showed an overall mortality rate of 34.5% after a median follow up of 27 days. Significant predictors of death were the active disease and age ≥70 years [29]. In the USA, for comparison, 8755 patients were diagnosed with MCL between 2000 and 2013. Incidence (MCL cases/100,000 persons) increased from 0.711 in 2000–2006 to 0.800 in 2007–2013 (*p* < 0.001), and was significant among older male and female patients ≥ 65 years, and among non-Hispanic Whites and Hispanics, but not among those aged <65 years and non-Hispanic Blacks [30,31].

Research continues, focused particularly on relapsed and high-risk patients. There are different treatment options for younger patients without comorbidities and for elderly fit patients [32]. High-dose therapy and autologous stem-cell rescue and high-dose cytarabine in younger patients has shown benefits, as has maintenance rituximab and bendamustine in older patients. Allogeneic stem cell transplant represents a potentially curative option for younger patients, but guidelines in 2017 stated that there were insufficient data to support this in front-line treatment [8,9,33]. The inclusion of ibrutinib, lenalidomide, and bortezomib represents an important advance for patients ineligible for, unable to tolerate, or failing high-intensity combination chemotherapy. However, effective therapy for patients with relapsed MCL with ibrutinib resistance, aggressive form of the disease, and/or transplant ineligible patients represents an important unmet medical need [8,34]. Minimal residual disease (MRD) has been increasingly investigated in MCL [35]. The potentially valuable use of MRD as a surrogate end point for progression-free survival in comparing the efficacy of different treatments in randomized trials is also still hampered by limitations in take-up in clinical routine, partly because of the need for patient-specific primers and standardization [36]. In contrast to real-time quantitative polymerase chain reaction (qPCR) of immunoglobulin heavy chain (IGH) or BCL1-IGH clonal markers, droplet digital PCR (ddPCR) allows absolute quantification of MRD samples. The European MCL network evaluates the prognostic significance of ddPCR in the context of prospective trials [37]. 

The value of the wider range of treatment options is not to be underestimated, but their optimal use requires fulfilment of a series of complementary conditions in testing, infrastructure, and training in advanced techniques. There is a need for a better understanding of how to incorporate novel therapies and utilization of risk-adapted treatment approaches together with a high unmet need for effective treatments, particularly for relapsed/refractory MCL and for elderly and frail patients [8,38]. The use of autologous anti-CD-19 CAR-T cell therapy, first authorized in Europe in 2020 [39], requires careful work-up of the patient, an experienced interdisciplinary team, and a specialized hospital with follow-up resources, as well as agreement on the balance of quality and efficiency, cost, access, and expertise to successfully implement this technology in healthcare [8,14,40]. Another challenge is that centers providing cellular therapy need a robust clinical infrastructure to handle the complex scheduling logistics, maintain the “chain-of-custody” and “chain-of-identity” of the cellular product, and facilitate communication to manage potentially severe toxicities. High costs can be associated with managing adverse events occurring during treatment. There is also a great need for highly qualified personnel and experienced clinical and non-clinical staff at the center [41]. The introduction of personalized medicine into clinical practice would permit a more precise response to the biological and clinical heterogeneity of MCL. However, that in turn also requires reliable prognostic tools [42,43,44,45].

### Common Problems through National Perspectives

The situation across Europe varies widely from country to country in terms of governance, clinical standardization, awareness and education, provisions for reimbursement, infrastructure, and evidence generation, although the challenges presented by MCL are similar everywhere. There is a lack of availability of modern diagnostic techniques—including p53 sequencing—and of novel treatments that have been approved by EMA, but remain restricted, often accessible only through clinical trials, and reimbursement for maintenance therapy is hard to obtain in some countries. Challenges in terms of infrastructure include deficient or absent biobanking of blood and marrow for MCL patients, limited availability of population-based data or information on local treatment patterns and outcome, and absence of reliable validated tools to assess mutational status.

This report provides a snapshot of varying conditions in a large sample of European countries represented and reported on in the EAPM expert panel, broken down into the broad categories of demand and supply. The categories are by nature somewhat arbitrary, and the data do not always fit precisely the sub-categories, but it offers an overview of some of the principal challenges in each country (see Appendix B). 

## 3. Demand

### 3.1. Governance

Given the challenges posed by MCL as a very rare cancer, with patients often presenting very aggressive forms of the disease, it would be important for these patients to benefit from the expertise of multidisciplinary teams as the model provided for by the European Reference networks. These have a vital role in developing understanding, diagnosis, and treatment of rare cancers, and their unique capacity to mobilize expertise across Europe through virtual collaboration is realizing much of what has been previously a scattered and therefore under-exploited asset. Moreover, given the variety of lymphoma subtypes, it is crucial to encourage the collection of clinical data on each sub-type of rare and ultra-rare cancers. However, there are continuing national variations in practice over prescribing, organization of registries and of hematological and transplant centers, and attention to determinants or potential risk factors [45]. The Lymphoma Study Association (LYSA) is an independent network dedicated to clinical lymphoma research projects, frequently in collaboration with international scientific teams. Its clinical studies range from first-in-man tests of new treatments to the establishment of reference therapeutic strategies [46]. The European MCL Network of 15 national lymphoma study groups complemented by experts in histopathology and molecular genetics established two standard treatments. For younger patients, it was Ara-C containing induction followed by Ara-C containing myeloablative consolidation with autologous stem cell rescue and rituximab maintenance. For elderly MCL patients, it was R-CHOP induction followed by rituximab maintenance [47] (see Appendix A).

### 3.2. Clinical Standardization 

There are different treatment options for patients with MCL, but most patients will start with chemoimmunotherapy. Stem cell transplants and radiation therapy are sometimes used in combination with chemoimmunotherapy. Additionally, CAR T-cell therapy was recently approved by the FDA for patients who have not responded to or have relapsed following treatment. With ongoing clinical trials, there is a great promise for new advancements [48]. Guidelines and recommendations have emerged at national and at European level, but sporadically rather than systematically, and they are not always followed, with national practices continuing to vary in implementing important biological risk factors in the clinical routine, treatment efficacy varies depending on histological lymphoma subtypes, and knowledge of factors associated with response to treatment or survival is currently still limited [45]. Consensus has yet to be reached regarding use of biomarkers for therapeutic decision-making in real-life settings (see Appendix A).

### 3.3. Awareness and Education

When first diagnosed with MCL, 30% of respondents did not understand the characteristics of their subtype. Furthermore, when it came to understanding side effects, 21% did not understand and the number was even higher (27%) of those who did not understand how to manage those side effects. These numbers prove the value of better communication between doctors and patients. Both healthcare providers and patients need to be aware that active communication is a key element during the treatment process, even more given the nature of MCL progression and the relapse rates [7]. Studies suggest that communication between peers and evidence-based and regularly updated national recommendations can significantly improve the outcomes of patients with lymphomas, even without the broad use of new expensive agents, and there is an appetite for collaboration and development of common standards, manifested for instance in regular meetings among regional experts in some countries (see Appendix A).

## 4. Supply

### 4.1. Reimbursement

MCL is one of the most aggressive lymphomas and can lead to poor patient outcomes. While targeted therapies are approved for MCL, reimbursement in many countries is still lacking or not provided for all components in the treatment regimen. With financial concerns being one of the top barriers to treatment, these patients may be confined to conventional therapies [7]. There is a split between hospital and other healthcare budgets—for basic or specialized drugs or transplant procedures—and distinct rules on prescribing authority or drugs from a list of expensive drugs for strictly defined indications. Physicians in some countries can administer drugs outside of the reimbursable list only with the agreement of hospital drug boards and management, and in others, there are strict limits and frequent delays on reimbursement of many newer therapies (see Appendix A).

### 4.2. Infrastructure

There have been many advances in understanding the biology of MCL, but centers that provide cellular therapy require a robust clinical infrastructure, and this poses a great challenge. Moreover, highly qualified personnel and experienced clinical and non-clinical staff are needed in centers [8]. There is a lack of adequate tissue banks for MCL, often owing to lack of dedicated funds and staff, and the absence of binding regulation, limited availability of advanced laboratories and more complicated molecular testing techniques such as p53 sequencing, and a lack of biobanks (see Appendix A). 

### 4.3. Evidence Generation

MCL is one of the most aggressive lymphomas and can lead to poor patient outcomes. Subtype reporting can be effective in providing specific unmet needs of patients with MCL [7]. However, countries suffer from insufficient provision for independent clinical studies, because of complicated bureaucracy and the lack of study infrastructure, the legislation covering biobanking and rules about contracting, and difficulties in obtaining new drugs, as well as human resources and laboratory logistics, and cost obstacles. Trials in early phases tend to be conducted more in the US, which is preferred due to the speed of approval there (see Appendix A).

## 5. Discussion

The availability of hematology centers varies, as does the existence of national treatment guidelines and recommendations, and specific issues such as consensus regarding use of biomarkers for therapeutic decisions. What is often encountered when clinical studies with CART therapy are set up is the lack of referral networks/mechanism between centers with infrastructure for CART and those without. Effective networking could help improving outcomes and this could be incentivized by the healthcare systems. Despite the differences from country to country, what also emerges is a range of common features. 

However, some of the common features reported offer a more optimistic perspective. There are numerous examples of national networks for diagnosis, treatment, or research, and frequent expressions of interest from clinicians and academics in alignment and cooperation—including at European level. A Croatian comparative study suggested communication between peers and evidence-based and regularly updated national recommendations can significantly improve the outcomes of patients with lymphomas, even without the broad use of new expensive agents [49]. The leading Spanish network of treatment centers ensures regular contacts and comparisons across the country. At European level, the ESMO Clinical Practice Guidelines for diagnosis, treatment and follow-up for MCL released in mid-2017 in accordance with the ESMO standard operating procedures for Clinical Practice Guidelines development was recently endorsed by the EHA Lymphoma Working Group (LyG) as a leading example of this form of collaboration [50].

The evident enthusiasm for better and more detailed diagnosis and treatment could find another outlet in pursuing further opportunities at the European level. Funding programs exist for actions addressing cancer—such as EU4Health, which published two waves of calls in 2021, leading to 16 major initiatives. The Work Program for 2022 was recently adopted and included again a significant number of actions addressing cancer, this time with a strong focus on prevention and diagnosis [51]. The EU’s Beating Cancer Plan is now coming on-stream, offering the possibility to shape its attention—and funding—to MCL. The evolving Cancer Mission also provides a chance to influence the EU’s strategic approach to different forms of cancer. The European Health Data Space proposed in early April 2022 aims to facilitate the data exchange among healthcare professionals, researchers, and innovators, and will offer funding for strengthening national as well as European data infrastructures [52]. In addition, the current plans for a revision of the EU’s general pharmaceutical legislation and the ongoing implementation of the EU’s recently adopted Health Technology Assessment regulation are opening up the discussion of access to new medicines in ways that could prove beneficial to all types of cancer. The key to any successful strategy at European level is mobilizing the maximum degree of national synergy in advance, so as to maximize the impact of messages delivered into the European decision-making arena.

## 6. Conclusions

While the optimal diagnosis and treatment of MCL is still disparate in Europe among population groups and between countries, a clearer identification of the gaps can help to plan continued improvements, at national and at European level. The awareness of the current deficiencies, coupled with the growing evidence of a will for a more coordinated and better-aligned approach, provides grounds for generating a common approach to policymakers at national and European level in order to obtain better support for the investment and services for physicians, researchers, and ultimately, patients. 

## Data Availability

Not applicable.

## Appendix A

### Challenge-by-Challenge Account

(a) Demand

1. Governance

In the Nordic countries, public health care operates on uniform criteria, with in general more liberal criteria for off-label drug use [53].

Three French registries are devoted to hematological malignancies and are subject to regular certification and quality control [28,54]. Recent improvements in patient survival in major lymphoma subtypes at population level raise new questions about patient outcomes such as quality of life or long-term sequelae [55]. Only a few epidemiological studies have addressed the extent to which socioeconomic status, social institutional context (i.e., healthcare system), social relationships, environmental context (exposures), individual behaviors (lifestyle) or genetic determinants influence lymphoma outcomes, especially in the general population.

In Portugal, there are 15 hematology centers accredited by the national hematology board and a hematology reference network was set up in 2016. There are six transplant centers, and there is national public healthcare coverage.

2. Clinical standardization

In Croatia, national recommendations exist for MCL treatment. MCL clinical guidelines were developed in Spain in 2022.

In the Nordic countries, the challenges are in implementing important biological risk factors in the clinical routine, including TP53 mutational status and histology.

In France, treatment efficacy varies greatly depending on histological lymphoma subtypes, and knowledge of factors associated with response to treatment or survival is currently still limited. Substantial developments have been made in the research of prognostic markers in relation with lymphoma pathogenesis, but there is currently no consensus regarding use of these biomarkers for therapeutic decisions in real-life settings.

In Belgium, since the publication of the guidelines by The Belgian Hematological Society Lymphoproliferative Working Party (BHS WM) in 2015, major changes occurred in the diagnostic work up and treatment. The BHS WM updated in 2018 the existing recommendations on best strategies for frontline and subsequent line treatment of MCL bases on new reimbursements and robust clinical data [56,57].

In Switzerland, studies have identified the limitations of the currently available consolidation and maintenance approaches for MCL patients and emphasized the need for rapid access to CAR-T cell treatment [58].

In Italy, there is inadequate awareness of options for maintenance therapy.

In the Czech Republic, outcomes have improved over the last two decades, mainly due to rituximab—median OS has been doubled (R of death reduced by 50%). There has been improvement in the last decade as well as in younger and older populations. A Czech lymphoma study group has six centers reflecting broadly the distribution of NHL patients. Guidelines updated in 2021 establish therapy standards for patients eligible and non-eligible for transplants, and treatment regimes for patients with early relapse and late relapse [59].

Treatment challenges in Portugal include limited information on local treatment patterns and outcomes, ensuring wider compliance with ESMO guidelines on regimens, more training/education, and reinforcement of reference networks.

3. Awareness and education

A Croatian comparative study suggested communication between peers and evidence-based and regularly updated national recommendations can significantly improve the outcomes of patients with lymphomas, even without the broad use of new expensive agents [49].

In Poland, the need is felt for collaboration within the European MCL network and development of common standards.

At present, 102 Spanish hospitals collaborate with the Geltamo network for lymphoma, and there is at least one person responsible for lymphoma in each hospital [60]. Two general meetings are organized with all the delegates and several educational activities are performed each year, some of them addressed to young hematologists.

(b) Supply

1. Reimbursement

In Croatia, hospital budgets pay for most drugs—but not transplant procedures and drugs from a list of expensive drugs for strictly defined indications. Physicians can administer drugs outside of the reimbursable list only with the agreement of hospital drug boards and management. Reimbursement delays jeopardize early availability of new drugs.

In the Nordic countries, there are diverse criteria for reimbursement for BTK inhibitors. Ibrutinib is available for MCL in Denmark and Finland, but not in Norway, and in Sweden only as a bridge to allogeneic transplant. Brexucabtagene autoleucel (CAR-T for MCL) is not reimbursed in any of the Nordic countries [61].

In Poland, treatment with BTK inhibitors and lenalidomide is not reimbursed, neither CAR-T cell therapy is reimbursed despite CAR-T for B-ALL in children and for DLBCL in adults now being covered by the specific reimbursement program. However, patient accessibility to this program is very limited due to monopolized and restrictive hospital certification process by pharmaceutical companies.

Spain faces lack of access to appropriate therapies and expertise, notably the delay between EMA approval and reimbursement of new therapies.

Switzerland has started decentralized manufacturing and providing CAR T cell therapies at 150,000 to 200,000 USD, approximately half the price of FDA-approved CAR T cell therapies in the USA [41].

Italy has staged reimbursement of CAR T-cell therapy, with payments made in three instalments linked to individual patient outcomes [41]. A strong forum is needed for discussions with regulators on easier access to drugs and allowing best treatment allocation. Currently, physicians are limited in possibilities of requesting approval.

In the Czech Republic, many of the treatments are not reimbursed and available only with special approval. Allo-SCT is reimbursed but is used very rarely. Ibrutinib does not still have permanent reimbursement, and there are long delays in granting reimbursement of new products [62].

In Portugal, there is limited access to novel agents and cell therapies outside trials without special authorization.

2. Infrastructure

All major hospitals in Croatia have a hematology unit, and ASCT is performed in five of them. AlloSCT and CAR-T cell therapy is performed in one hospital. Collaboration is good and referrals are easy. The Croatian Hematologic Society and Croatian Cooperative Group for Hematologic Diseases defined 6 designated hematopathology centers [49]. Diagnostics in Croatia have improved in the last decades with the designation of referral hematopathology centers, improved immunohistochemistry, and increased availability of FISH and flow cytometry. However, there is limited availability of more complicated molecular testing techniques such as p53 sequencing, and a lack of biobanks and advanced laboratories.

Poland suffers from a lack of adequate tissue banks for MCL, owing to lack of dedicated funds and staff, and the absence of binding regulation. In Poland, most hematological centers correctly diagnose MCL. Imaging diagnostics are mostly CT scans in older patients with PET-CT examination reserved for younger patients and assessment before and post-ASCT.

The Spanish registry for lymphoma and chronic lymphoproliferative disorders RELINF has been functioning since 2014 and covers more than 18,000 patients. Late or incorrect diagnosis is not usually a challenge in Spain, but there exists the need for development, validation, and implementation of biomarkers (prognostic and or predictive) in clinical practice.

Italy needs to boost diagnosis (such as with TP53) on a national basis to reach smaller centers.

In the Nordic countries, greater quality assurance is needed in pathology when assessing histology.

In the Czech Republic, what needs improvement is biobanking and the dissemination of modern diagnostic techniques.

Morphology/immunohistochemistry (including Cyclin D1) is available in most centers in Portugal, though some send samples out; flow cytometry is universally available, FISH available in the main centers, and diagnostic accuracy is adequate, but with delays in peripheral centers. A pathology reference network was created in 2016 but its use is not mandatory for centers. Gene expression studies and sequencing are not usually performed. Investment is needed in referral centers—mostly human resources and infrastructure, equipment needs are limited. That will allow support and networking, training, and education of other professionals. Other outstanding needs are for biobanking, human and technical resources, training, and infrastructure in the research setting.

3. Evidence generation

There is insufficient provision in Croatia for clinical studies, because of complicated bureaucracy and the lack of study infrastructure. (CTs are also felt by some to have been “hijacked by pharma companies” in a small country with a limited patient pool and a high level of routinely available health care.)

In Poland, academic clinical trials face difficulties in obtaining new drugs, systemic problems of human resources and laboratory logistics, as well as cost obstacles. However, commercial trials are seen as imposing treatment options, employing eligibility criteria that are often difficult to meet, time constraints, and recruitment pressure.

In Spain, the Spanish lymphoma group GELTAMO has been functioning since 1990 and runs research studies and clinical trials. GELTAMO also participates in clinical trials and studies proposed by other European cooperative groups. Specific challenges exist in terms of the lack of commercial feasibility in developing new therapies. Developing treatments still depends largely on the pharma industry. There is an increasing difficulty in generating independent data from cooperative groups because of funding and regulatory issues. Even for observational studies, retrospective and prospective, resources are a limiting factor. New regulations in Spain for observational studies with drugs impose controls similar to a clinical trial. The lack of tissue banks is another challenge and a critical aspect for biomarker discovery and validation. Clinical trials should incorporate the collection of samples to allow translational studies, ideally in cooperative groups. The main issues are the funding, legal aspects, and informed consent.

The Nordic lymphoma group in Sweden, Norway, Denmark, and Finland conducts CTs, and gains from taking a Scandinavian perspective of the joint 25 million population by access to national lymphoma registries for real-world data. However, there is also bureaucracy in clinical trials development relating both to the legislation covering biobanking and rules about contracting.

In France, most lymphoma patients are not included in clinical trials due to stringent inclusion criteria. In addition, patients above a certain age, with comorbidities or already receiving some medications are most often excluded.

UK data support the now established use of ibrutinib as a bridge to consolidation with allo-SCT in younger fitter patients. Chimeric antigen receptor-T cell therapy trials after BTKi failure are also showing promising results [63].

The Fondazione Italiana Linfomi develops research projects in the lymphoma field, independently or in collaboration with major study groups conducting international projects, enrolling more than 1000 patients a year in some 90 clinical studies at the network of 90 onco-hematology research centers, benefiting from advice from 12 scientific boards covering different lymphoma aspects. It promotes non-profit research projects independent of pharma companies [64].

The Portuguese lymphoma group aims to use the national cancer registry to access epidemiological data on lymphoma to establish a network of associated pathologists so as to improve standards for diagnosis, to conduct retrospective studies and analysis of clinical practices, and to conduct biological and correlative studies and prospective studies. Research needs include access to biomarkers, better access to MRD determination (currently only with limited availability) and to molecular studies (particularly TP53 mutations). Clinical research challenges include raising the current low level of activity, improving networking and physician/centers’ motivation, and organizational, training, and human resources, alongside funding.

## Appendix B

### Country-by-Country Account

In Croatia, national recommendations exist for MCL treatment. Hospital budgets pay for most drugs—but not transplant procedures and drugs from a list of expensive drugs for strictly defined indications. Physicians can administer drugs outside of the reimbursable list with the agreement of hospital drug boards and management. Reimbursement delays due to national financial problem jeopardize early availability of new drugs. All major hospitals have a hematology unit, and ASCT is performed in five of them. AlloSCT and CAR-T cell therapy is performed in one hospital. Collaboration is good and referrals are easy. The Croatian Hematologic Society and Croatian Cooperative Group for Hematologic Diseases defined 6 designated hematopathology centers. Moreover, there is insufficient provision for clinical studies, because of complicated bureaucracy and the lack of study infrastructure (CTs are also felt by some to have been “hijacked by pharma companies” in a small country with a limited patient pool and a high level of routinely available health care).

The number of new MCL patients in the National Research Institute of Oncology in Poland has been rising gradually since 2016. Currently, most hematological centers correctly diagnose MCL. Imaging diagnostics are mostly CT scans in older patients with PET-CT examination reserved for younger patients and assessment before and post-ASCT. Treatment with BTK inhibitors and lenalidomide or CAR-T cell therapy is not reimbursed. Academic clinical trials face difficulties in obtaining new drugs available, systemic problems of human resources and laboratory logistics, and cost obstacles. However, commercial trials are seen as imposing treatment options, employing eligibility criteria that are often difficult to meet, time constraints, and recruitment pressure. The country suffers a lack of adequate tissue banks for MCL, owing to lack of dedicated funds and staff, and the absence of binding regulation. There is a need for collaboration within the European MCL network and development of common standards

In Spain, the Spanish lymphoma group GELTAMO has been functioning since 1990 and runs research studies and clinical trials. The Spanish registry for lymphomas and chronic lymphoproliferative disorders has been functioning since 2014 and covers more than 18,000 patients. MCL accounts for just 5% of cases reported. At present, 102 Spanish hospitals collaborate with the GELTAMO network for lymphoma, and there is at least one person responsible for lymphoma in each hospital [60]. Two general meetings are organized with all the delegates. Several MCL clinical trials are promoted by cooperative groups in Spain. MCL clinical guidelines have been developed in 2022. Late or incorrect diagnosis is not usually challenge, but there exists the need for development, validation, and implementation of biomarkers (prognostic and or predictive) in clinical practice. There is also the challenge of the lack of access to appropriate therapies and expertise, notably the delay between EMA approval and reimbursement of new therapies. Specific challenges in terms of the lack of commercial feasibility in developing new therapies. Developing treatments still depends largely on the pharma industry. The use of rituximab is off label in mantle cell, but its use in maintenance is usually allowed. There is an increasing difficulty in generating independent data from cooperative groups because of funding and regulatory issues. Even for observational studies, retrospective and prospective, the amount of resources needed is limiting. New regulations in Spain for observational studies with drugs impose controls similar to a clinical trial. The lack of tissue banks is another challenge and a critical aspect for biomarker discovery and validation. Clinical trials should incorporate the collection of samples to allow translational studies, ideally in cooperative groups. The main issues are the funding, legal aspects, and informed consent.

The Nordic lymphoma group in Sweden, Norway, Denmark, and Finland conducts CTs, and gains from taking a Scandinavian perspective of the joint 25 million population by access to national lymphoma registries for real-world data. Public healthcare operates on uniform criteria and liberal criteria for off-label drug use. The challenges are in implementing important biological risk factors in the clinical routine, and greater quality assurance is needed in pathology when assessing histology. There is also bureaucracy in clinical trials development relating both to the legislation covering biobanking and rules about contracting. Trials in early phases tend to be conducted more in the US which is preferred due to the speed of approval there.

In France, lymphoma incidence rates continue to rise but there is limited data on MCL in France. Three French registries are devoted to hematological malignancies and are subject to regular certification and quality control. Other outstanding needs are for biobanking, and human and technical resources, training, and infrastructure in the research setting.

Recent improvements in patient survival in major lymphoma subtypes at population level raise new questions about patient outcomes such as quality of life or long-term sequelae. Only few epidemiological studies have addressed the extent to which socioeconomic status, social institutional context (i.e., healthcare system), social relationships, environmental context (exposures), individual behaviors (lifestyle), or genetic determinants influence lymphoma outcomes, especially in the general population. Treatment efficacy varies greatly depending on histological lymphoma subtypes, and knowledge of factors associated with response to treatment or survival is currently still limited. Substantial developments have been made in the research of prognostic markers in relation with lymphoma pathogenesis, but there is currently no consensus regarding use of these biomarkers for therapeutic decisions in real-life settings. Most of the knowledge on disease behavior and treatment efficacy comes from clinical trials. However, most lymphoma patients are not included in clinical trials due to stringent inclusion criteria. Moreover, patients above a certain age, with comorbidities or already receiving some medications are most often excluded.

UK data support the now established use of ibrutinib as a bridge to consolidation with allo-SCT in younger fitter patients. Chimeric antigen receptor-T cell therapy trials after BTKi failure are also showing promising results.

The Belgian Hematological Society Lymphoproliferative Working Party updated in 2018 the existing recommendations on best strategies for frontline and subsequent line treatment of MCL based on new reimbursement decisions and clinical data [56].

In Switzerland, studies have identified the limitations of the currently available consolidation and maintenance approaches for MCL patients and emphasized the need for rapid access to CAR-T cell treatment. Switzerland has started decentralized manufacturing and providing CAR T cell therapies at 150,000 to 200,000 USD, approximately half the price of FDA-approved CAR T cell therapies in the USA [65].

Italy has staged reimbursement of CAR T-cell therapy, with payments made in three instalments linked to individual patient outcomes. The Fondazione Italiana Linfomi develops research projects in the lymphoma field, independently or in collaboration with major study groups conducting international projects, enrolling more than 1000 patients a year in some 90 clinical studies at the network of 90 onco-hematology research centers, benefiting from advice from 12 scientific boards covering different lymphoma aspects. It promotes non-profit research projects independent of pharma companies. Italy needs to boost diagnosis (such as with TP53) on a national basis to reach smaller centers. It is felt to be important to stress the need not just to diagnose but to characterize the disease. A strong forum is needed for discussions with regulators on easier access to drugs and allowing best treatment allocation. Currently, physicians are limited in possibilities of requesting approval. Change of treatments will be the principal method to improve patient outcomes. There is inadequate awareness of options for maintenance therapy.

In the Czech Republic, prevalence has increased but outcomes have improved over last two decades, mainly due to rituximab—median OS has been doubled (R of death reduced by 50%). There has been improvement in the last decade as well as in younger and older populations. A Czech lymphoma study group has six centers reflecting broadly the distribution of NHL patients. Guidelines updated in 2021 establish therapy standards for patients eligible and non-eligible for transplants, and treatment regimes for patients with early relapse and late relapse. However, many of the treatments are not reimbursed and available only with special approval. Allo-SCT is reimbursed but is used very rarely. What needs improvement is biobanking, the dissemination of modern diagnostic techniques, faster drug availability (ibrutinib does not still have permanent reimbursement), and fewer limitations on reimbursement so that it becomes almost automatic one a product has EMA approval.

In Portugal, there are 15 hematology centers accredited by the national hematology board and a hematology reference network was set up in 2016. There are six transplant centers, and there is national public healthcare coverage. The Portuguese lymphoma group aims to use the national cancer registry to access epidemiological data on lymphoma, to establish a network of associated pathologists so as to improve standards for diagnosis, to conduct retrospective studies and analysis of clinical practices, and to conduct biological and correlative studies and prospective studies. Morphology/immunohistochemistry (including Cyclin D1) are available in most centers though some send samples out; flow cytometry is universally available, FISH available in the main centers, and diagnostic accuracy is adequate, but with delays in peripheral centers. A pathology reference network was created in 2016 but its use is not mandatory for centers. Research needs include access to biomarkers, better access to MRD determination (currently only with limited availability) and to molecular studies (particularly TP53 mutations). Gene expression studies and sequencing are not usually performed. Other outstanding needs are for biobanking, and human and technical resources, training, and infrastructure in the research setting. Treatment challenges include limited information on local treatment patterns and outcomes, ensuring wider compliance with ESMO guidelines on regimens, more training/education, and reinforcement of reference networks. There is limited access to novel agents and cell therapies outside trials without special authorization. Clinical research challenges include raising the current low level of activity, improving networking and physician/centers’ motivation, and organizational, training, and human resources, alongside funding. Participation is assured with European cooperative groups including EORTC. Investment is needed in referral centers—mostly human resources and infrastructure, equipment needs are limited. That will allow support and networking, training, and education of other professionals.
